# Galewone, an Anti-Fibrotic Polyketide from *Daldinia eschscholzii* with an Undescribed Carbon Skeleton

**DOI:** 10.1038/s41598-019-50868-9

**Published:** 2019-10-04

**Authors:** Ai Hua Zhang, Nan Jiang, Xing Qi Wang, Ren Xiang Tan

**Affiliations:** 1 Institute of Marine Biology, College of Oceanography, Hohai University, Nanjing, 210098 China; 2 Institute of Functional Biomolecules, State Key Laboratory of Pharmaceutical Biotechnology, Nanjing University, Nanjing, 210046 China; 3 School of Pharmacy, Nanjing Medical University, Nanjing, 210029 China

**Keywords:** Chemical biology, Medical research

## Abstract

A novel polyphenolic natural product, galewone, with undescribed carbon skeleton, was isolated as a racemate from the culture of Daldinia eschscholzii IFB-TL01, a fungus obtained from the mantis (Tenodera aridifolia) gut. The galewone structure was elucidated by a combination of MS and NMR spectra, and substantiated by X-ray crystallographic diffraction. The absolute stereochemistry of each galewone enantiomers was determined by the CD spectrum. In compliance of the structural similarities, galewone might be the shunt products of the dalesconol biosynthetic pathway. Both (−)- and ( + )-galewones were evaluated to be anti-fibrotic against activated hepatic stellate cell line, CFSC-8B, with the IC_50_ values being 3.73 ± 0.21 and 10.10 ± 0.41 μM, respectively. Thus, galewone may serve as a starting molecule for the discovery of new anti-fibrotic drug.

## Introduction

In liver, hepatic stellate cells function in the physiological condition to regulate retinoid homeostasis and remodel extra cellular matrix, by producing metalloproteases and their inhibitors as well as extracellular matrix components^1^. However, after chronic liver injury, hepatic stellate cells (HSC) become activated and characterized by increased proliferation, motility, overproduction of α-smooth muscle actin (α-SMA) and extracellular matrix proteins and losing retinoid^2,3^. As a result of chronic liver damage, HSC become myofibroblasts (MFB)-like cells which eventually develop into liver fibrosis^4–6^. Previous studies have shown that hepatic stellate cells, once activated, could play a considerable role in the development of liver fibrosis^7^. Thus, the compounds capable of eliminating the effects of activated hepatic stellate cells are beneficial for the liver fibrosis therapy. However, such an eliminating effect must be sufficiently selective because some severe side effects occur if the molecules have the same/similar effects on the quiescent hepatic stellate cells. This is why there remains a lack of a well-tolerated and efficient medicament that impedes the progression of liver fibrosis.

## Results

Owing to its production of a series of bioactive naphthol radical polymerized metabolites with undescribed architectures, the mantis-associated fungus *Daldinia eschscholzii* IFB-TL01 has attracted remarkable attention^8–13^. As part of our continuing research on discovery of anti-fibrotic metabolites, the EtOAc extract of the *D. eschscholzii* culture was selected for investigation. As a result, the new naphthol derivative, galewone, was characterized and demonstrated to possess a promising potential in anti-fibrotic action. Herein, we present the structure elucidation, bioactivity evaluation, and plausible biogenetic pathway of galewone.

**Figure 1:**
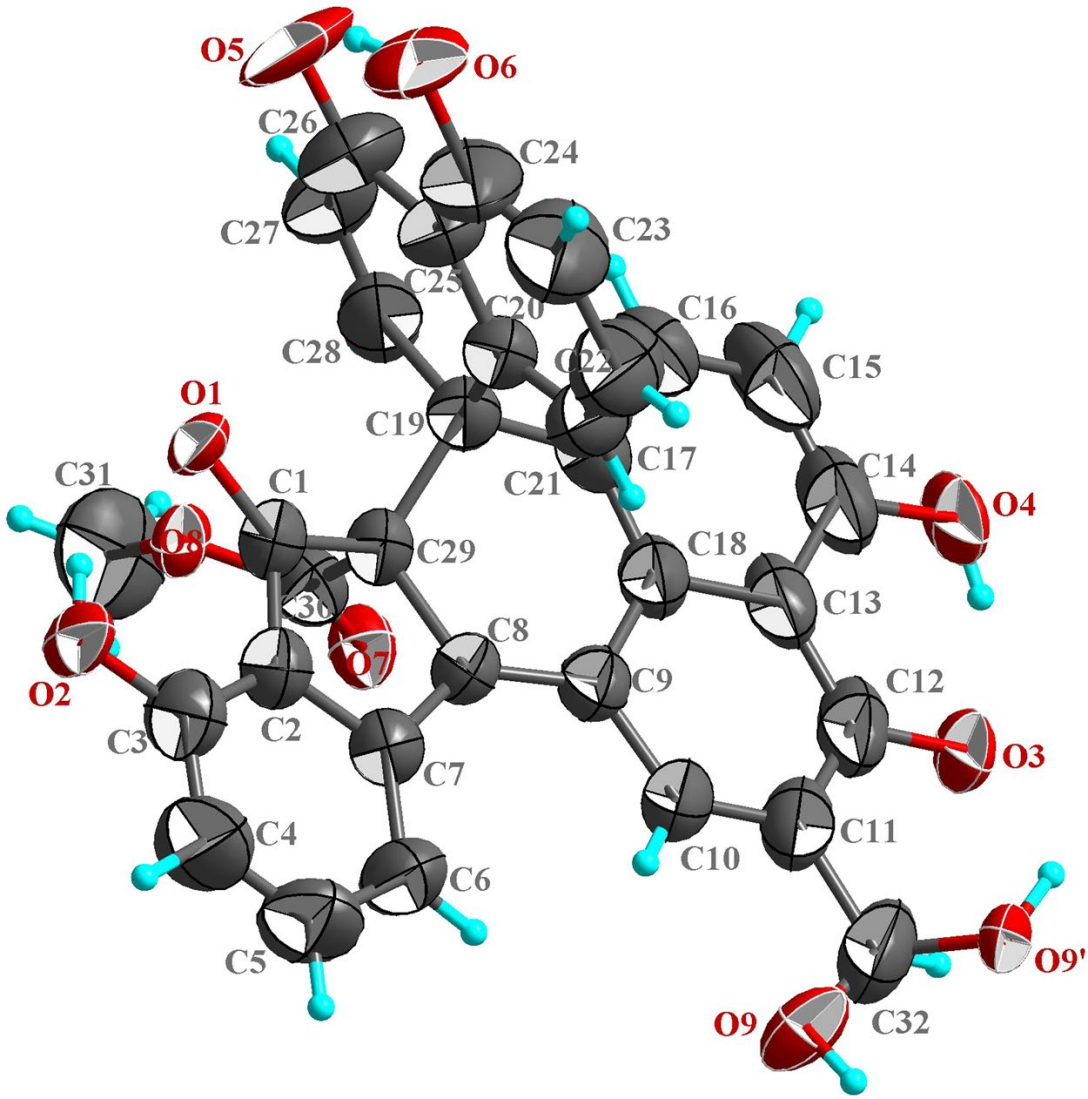
X-ray crystallographic structure of (±)-galewone.

**Figure 2:**
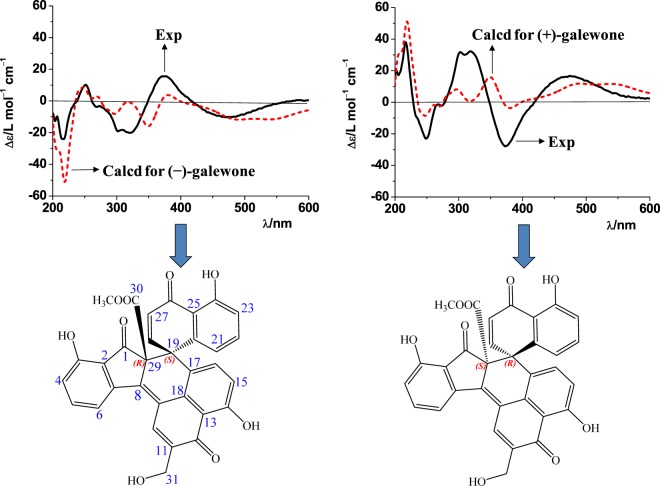
Absolute stereochemical assignments for (+)- and (−)-galewone by comparison of the recorded (solid lines) and computed (dotted lines) ECD spectra.

Galewone afforded as red needles, was ascertained to have a molecular formula of C_32_H_20_O_9_ from the sodium-liganded molecular ion at *m/z* 571.09910 (calcd. for C_32_H_20_O_9_Na, 571.09995) in its high-resolution electrospray ionization mass spectrometry (HR-ESI-MS). This observation, along with its 1D and 2D NMR spectra (Table S2 and Figs 6–11 suggested that it was probably hydroxymethylated spirodalesol^12^. The ^1^H NMR spectrum of galewone suggested the presence of two groups of *ortho-*coupled protons (δ_H_ 7.01/H-15 and 7.28/H-16; δ_H_ 6.94/H-27 and 7.48/H-28), two groups of three *ortho*-protons (δ_H_ 6.97/H-4, 7.68/H-5, and 7.75/H-6; δ_H_ 6.54/H-21, 6.99/H-22, and 6.77/H-23), one hydroxyethyl group (δ_H_ 4.85), one methoxyl group (δ_H_ 3.59), and three phenolic protons (δ_H_ 8.79/HO-3, 12.78/HO-14, and 12.70/HO-24). The ^13^C NMR and DEPT experiments evidenced the presence of 32 carbons grouped, including three carbonyl signals (δ_C_ 197.6/C-1, 189.6/C-12, and 189.8/C-26). The most possible structure of galewone was assigned via the HMBC correlations of the H-6 with δ_C_ 124.1/C-2 and 139.8/C-8, of the H-10 with δ_C_ 139.8/C-8, 189.6/C-12 and 132.3/C-18, and of the H-16 with δ_C_ 162.0/C-14, 132.3/C-18 and 47.5/C-19, and of the H-21 with δ_C_ 47.5/C-19, 117.8/C-23 and 115.1/C-25, and of the H-31 with δ_C_ 131.6/C-10 and 189.6/C-12. Subsequently, the novel skeleton and relative configuration of galewone were confirmed by its single crystal X-ray diffraction (Fig. 1). Since galewone was obtained as a racemic mixture, chiral HPLC separation was performed to give two enantiomers ( + )-galewone and (−)-galewone, which were disclosed to possess (19 *R*,29 *S*)- and (19 *S*,29 *R*)-configurations (Fig. 2), respectively, by comparing their CD curves with the ECD spectra computed for all optional stereoisomers. In the calculated curves, the first Cotton effect centered at 218 nm, arising from the electronic transition from filled C=C bonding molecular orbital (MO, *π*_*C=C*_) to the anti-bonding C-C orbital (${\sigma }_{C-C}^{\ast }$) (MO 135 → 146) (Figs 2, S4 and Table S4), corresponded to the peak at 218 nm in the acquired CD spectra. The next Cotton effect at 321 nm in the recorded CD spectra could be correlated to the shoulder peaks at 298 and 351 nm in the computed counterparts. The electronic transitions from *π*_*C=C*_ and the lone pair orbital of oxygen (*n*_*O*_) to the ${\pi }_{C=C}^{\ast }$ orbital contributed to these absorption bands (MO 140 → 145 and MO 137 → 143). In addition, the Cotton effects at 251 and 375 nm in the acquired CD spectra were also reproduced by the calculations at 236 and 374 nm, respectively. The electronic transitions from *π*_*C=C*_ to the ${\pi }_{C=C}^{\ast }$ orbitals contributed to these absorption bands (MO 133 → 145 and MO 142 → 145, Table S4).

**Figure 3:**
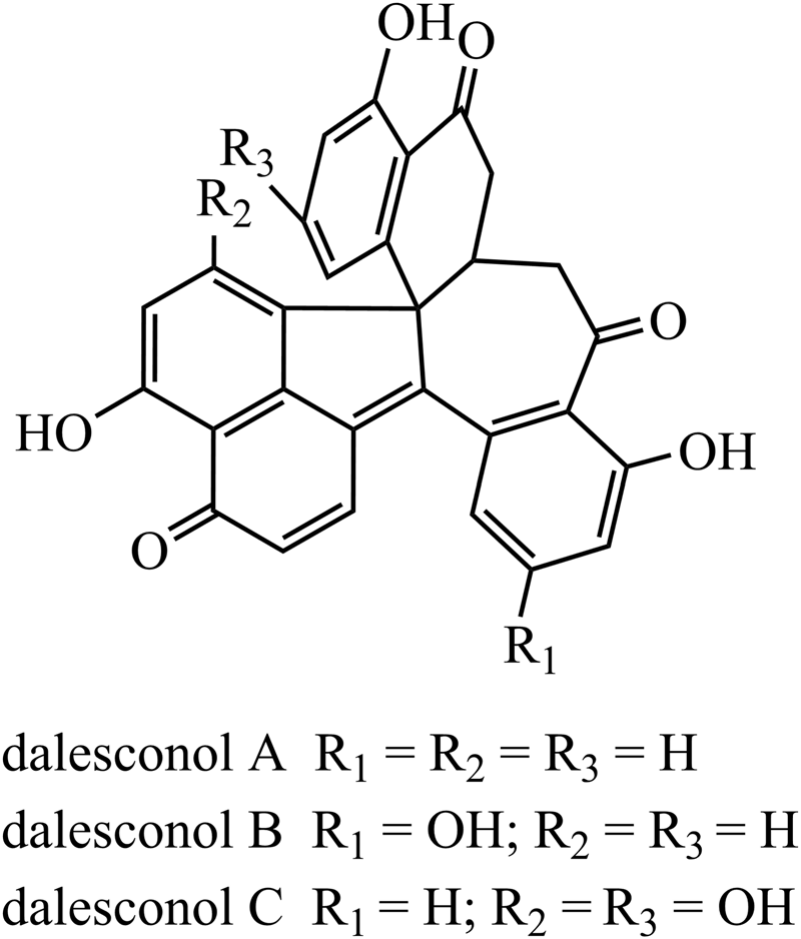
The structure of dalesconols A–C^8,10^.

**Figure 4:**
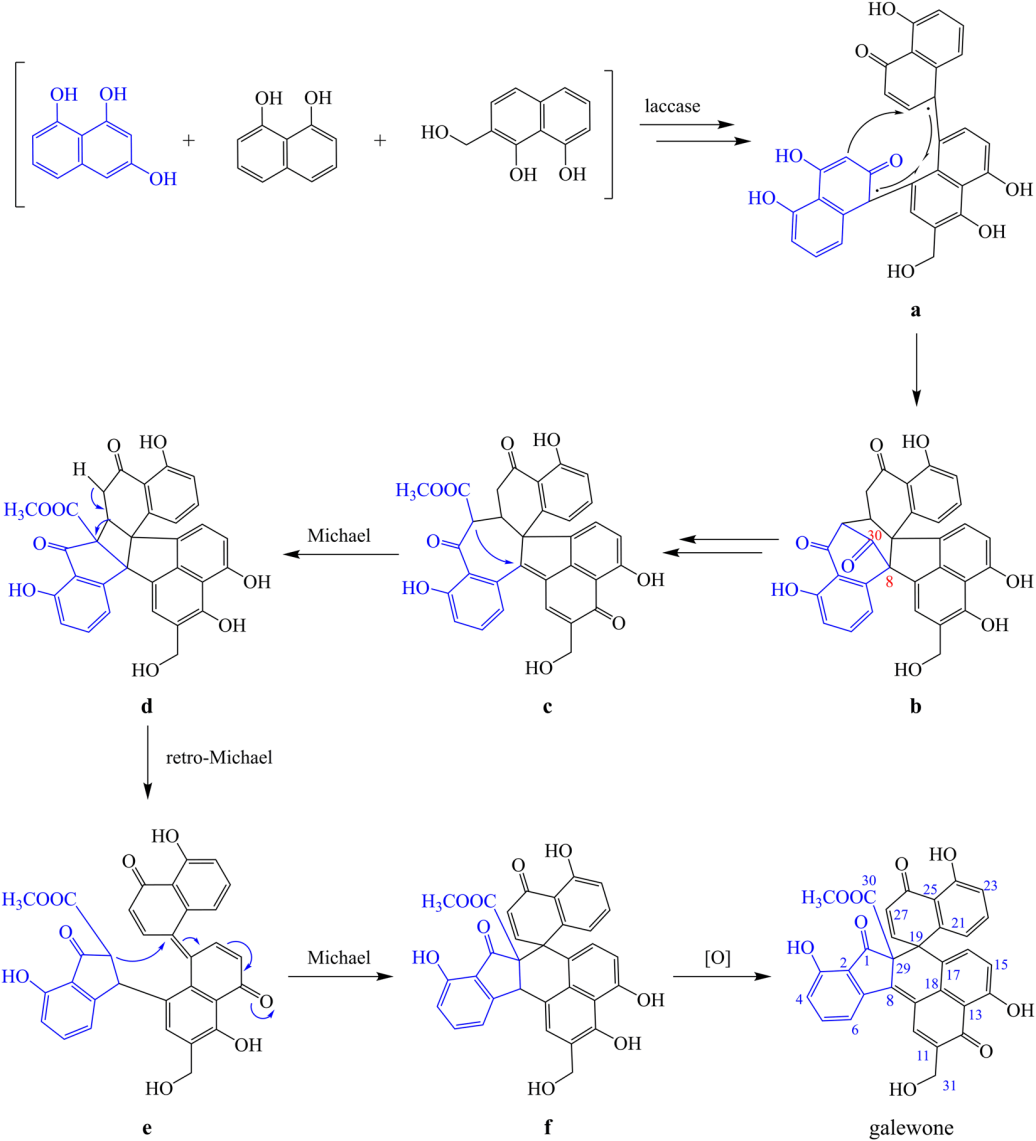
Plausible biosynthetic pathway of galewone.

With the absolute configuration of galewone enantiomers determined, we initiated their biosynthesis through probing into their possible naphthol precursors. The structural similarities between galewone and dalesconols suggested that they might share a similar mechanism to perform their biosynthesis^10^. Galewone embodied two sets of intramolecular hydrogen bonds between phenolic hydroxyl group and adjacent carbonyl group similar to those of dalesconols A–C (Fig. 3), together with that they each consisted of seven rings. The compounds obtained from six-acetyl-CoA^9^ provided the possible naphthol radicals, which possessed unique carbon chains and participated in the formation of galewone. For instance, the existence of key intermediate 2-(hydroxymethyl)-naphthalene-1,8-diol (Fig. 4) was rationalized by such related compound as 3,4,8-trihydroxy-6-(hydroxymethyl)-3,4-dihydronaphthalen-1(2 H)-one^9^. These facts demonstrated that galewone might originate from an similar biogenetic mechanism analogous to that of dalesconols, which initiated by the combination of three naphthol radicals (Fig. 4).

Single enantiomers and racemate of galewone were comparatively evaluated for the anti-fibrotic activities. CFSC-8B was incubated with (−)-, ( + )-, and (±)-galewone at concentrations from 0.1 to 30 μM, respectively. The cell viability of CFSC-8B cells^14,15^ was significantly inhibited by (−)-, (+)-, and (±)-galewone with the IC_50_ values determined to be 3.73 ± 0.21, 10.10 ± 0.41, and 10.90 ± 0.62 μM, respectively (Table S1). Interestingly, (−)-galewone showed a much weaker cell viability inhibition effect on Lx-2 cells which in quiescent phase (LX-2 cells cultured 3 days as quiescent cells^1,16,17^) with IC_50_ value of 26.60 ± 3.87 μM than that in CFSC-8B cells (Fig. S1b and Table S1). These results suggested that (−)-galewone selectively reduce the cell viability of activated hepatic stellate cells.

**Figure 5:**
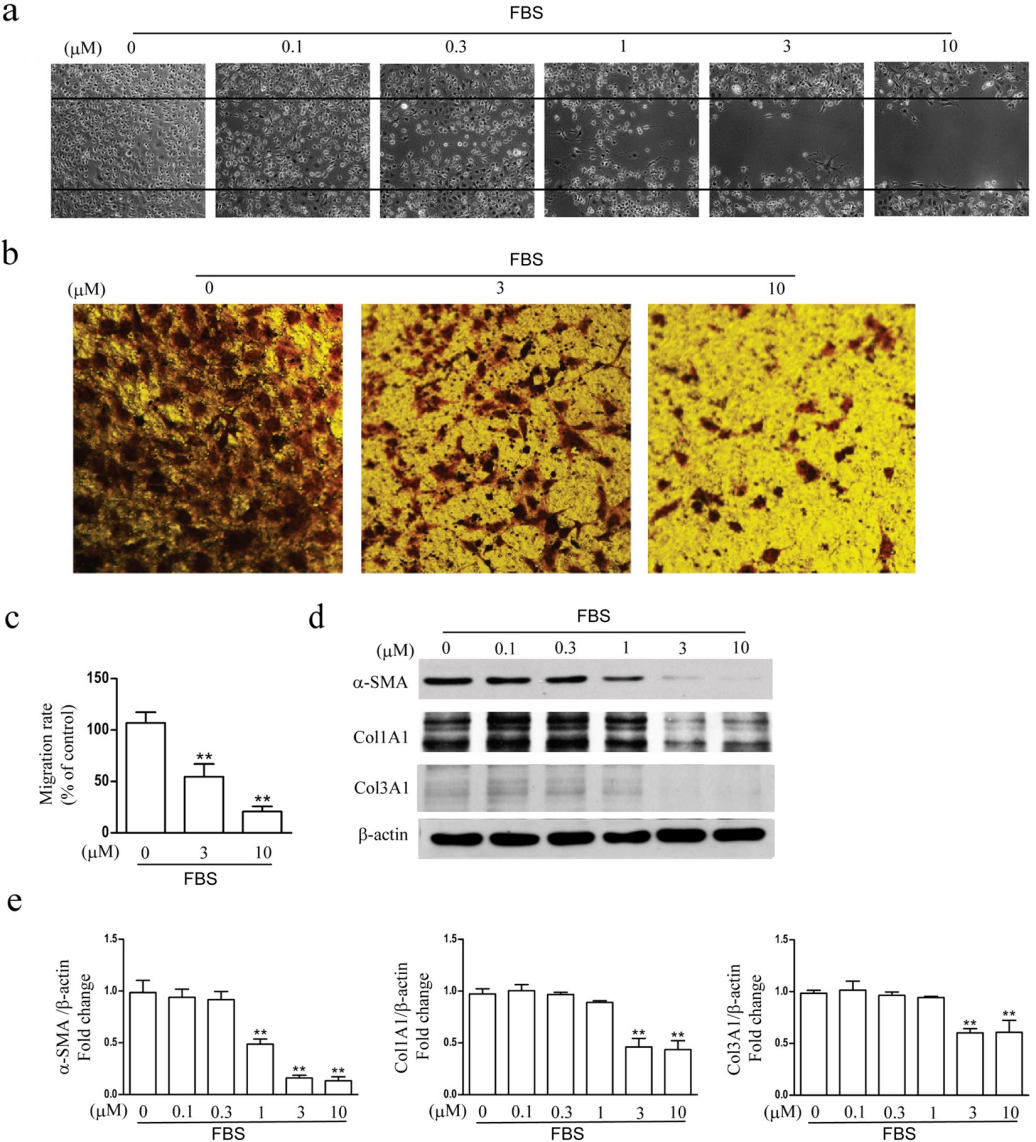
Effect of (−)-galewoneon CFSC-8B cells migration and expressions of α-SMA, Col1A1 and Col3A1 induced by FBS. (**a**) The migration capability was measured by wound-healing assay. (**b,c**) CFSC-8B cells that migrated to the lower compartment of invasion chamber were quantified by cell counting, and data were expressed as a percentage of vehicle group. (**d,e**) The expressions of the hallmark proteins of activated hepatic stellate cells induced by FBS includingα-SMA, Col1A1 and Col3A1 were analyzed by western blotting. Data were presented as means ± SEM. *P < 0.05, **P < 0.01 *vs*. vehicle group.

Activated hepatic stellate cells, such as CFSC-8B, have a strong migration capability and express some proteins aggravating liver fibrosis. Inhibition of the proliferation of CFSC-8B could prevent the migration and alleviate liver fibrosis. In our study, wound-healing and invasion chamber assay suggested that (−)-galewone blocked migration of activated hepatic stellate cells. In model group, FBS could induce wound closure 24 h after wound in gowning to CFSC-8B cells migration. However, activated hepatic stellate cells migration induced by FBS was dramatically and dose-dependently inhibited by (−)-galewone as indicated by larger the wounded area (Fig. 5a). Moreover, in BIOCOAT MATRIGEL chamber systems,10% FBS incubation with CFSC-8B cells induced migration of activated hepatic stellate cells was also significantly inhibited by (−)-galewone (Fig. 5b,c). Previous report suggested that activated hepatic stellate cells were responsible for the enhanced expression of α-SMA, Col1A1 and Col3A1^18,19^. As determined by Western bloting, (−)-galewone addition suppressed the expression of the marked proteins of CFSC-8B cells in a dose-dependent manner (Fig. 5d,e).

## Discussion

In conclusion, we have discovered a structurally intriguing metabolite from *Daldinia eschscholzii* IFB-TL01, and proposed a possible pathway for its formation. The naphthol radicals as unique starter units might be coupled with intermolecular logic participating in the construction of the neat sequential compartmentalization of unforeseeable seven ring frameworks, and this finding could set the foundation for further characterization of distinctive radical machineries to produce galewone analogues with more complex biological functionalizations by combinatorial biosynthesis. Specifically, the anti-fibrotic activities of (−)-, (+)-, and (±)-galewone were observed to reduce the cell viability of activated hepatic stellate cells, but to inhibit very weakly that of Lx-2 cells in quiescent phase (Table S1), indicating that galewone may serve as a starting molecule for the discovery of new anti-fibrotic drug.

## Experimental Section

### Ethics approval

Specific pathogen-free, 8–10-week-old female BALB/c mice were purchased from Experimental Animal Center of Jiangsu Province (Jiangsu, China). They were maintained with free access to pellet food and water in plastic cages at 25 ± 2 °C and kept on a 12 h light/dark cycle. Animal welfare and experimental procedures were carried out strictly in accordance with the Guide for the Care and Use of Laboratory Animals (National Institutes of Health, the United States) and the related ethical regulations of our university. All efforts were made to minimize animals’ suffering and to reduce the number of animals used.

### Statement

All methods were carried out in accordance with relevant guidelines and regulations, in addition all experimental protocols were approved by Nanjing University and Hohai University.

### General instrument and fungal Strain

As described^8,13^.

### Cultivation and metabolites isolation

*D. eschscholzii* IFB-TL01 was cultured on slants of potato dextrose agar (PDA) at 25 °C for 5 days. The fresh mycelium taken from the fungal colony in the Petri Dish was inoculated into the 1000 mL conical flasks each containing 300 mL of malt-extract medium (20 g/L malt extract, 20 g/L sucrose, 1 g/L peptone), and then the culture was shaken at 28 °C and 200 rpm/min for 3 days. The fermentation was repeated (6 × 300 mL) in order to inoculate into a 50 L fermentor containing 30 L of sterilized malt-extract medium. After a subsequent fermentation for 36 h, these were then seeded into scale-up cultures (350 L malt-extract broth in 500 L fermentor) at 25 °C and 100 rpm for 7 days (antifoam 0.1‰, aeration rate 8000 NL/h). The broth was collected and extracted with EtOAc, and a solid (302.7 g) obtained from *in vauo* evaporation of solvent of the extract was subjected to column chromatography (CC) over silica gel (1000 g, 300–400 mesh, 50 × 15 cm) eluted with CH_2_Cl_2_/MeOH mixtures (v/v 100:0, 100:1, 100:2, 100:4,100:8, 100:16, 100:32, 0:100). Further purification of the “100:4”-eluted CC fraction was accomplished by gel filtration over Sephadex LH-20 in MeOH, followed by HPLC separation to afford galewone (3 mg).

### Single crystal X-ray diffraction

The structure was solved by direct methods (SHELXS-97) and refined using full-matrix least-squares difference Fourier techniques. Crystallographic data in CIF format have been deposited in the Cambridge Crystallographic Data Centre [available free of charge at http://www.ccdc.cam.ac.uk/deposit or from the CCDC, 12 Union Road, Cambridge CB21EZ, UK; fax: (+44) 1223–336–033; or e-mail: deposit@ccdc.cam.ac.uk].

### Crystal data

Diffraction measurements were performed at 296 K on an Agilent Super Nova diffractometer equipped with Cu-*K*_α_ radiation (*λ* = 1.54178 Å). C_32_H_20_O_9_, *M*_r_ = 548.48, monoclinic, space group *P*2_1_/n, *a* = 9.1106 (2) Å, *b* = 20.9125 (5) Å, *c* = 13.1349 (3) Å, *V* = 2464.34 (10) Å^3^, *Z* = 4, *D*_*x*_ = 1.478 g/cm^3^, *μ* = 0.913 mm^−1^ and *F*(000) = 1136.0; crystal dimensions: 0.19 × 0.17 × 0.14 mm^3^; 3060 unique reflections with 4052 obeying the *I* > 2σ (*I*); *R*1 = 0.062, *wR*2 = 0.197, *S* = 1.013; supplementary publication no. CCDC-1043833.

### ECD calculation details

The density functional theory (DFT) at B3LYP/6−31 G (d, p) level was employed to optimize the geometries of the studied systems, taking crystal structures as the original configuration. The solvent effects on the electronic structures of the concerned systems were evaluated by quantum chemistry method through the polarizable continuum model (PCM, dielectric constant ε = 32.64 for CH_3_OH). Then, the corresponding excited-state calculations were performed at the ground-state optimized geometries. Time-dependent DFT in combination with PCM model (TD-DFT/PCM) with the same basis set was carried out to calculate the spin-allowed excitation energy and rotatory strength of the lowest 100 excited states. The UV and ECD spectra were generated using the program SpecDis by applying a Gaussian band shape with the width of 0.20 eV, from oscillator strengths and dipole-velocity rotational strengths, respectively. All the calculations were performed with the Gaussian09 program.

### Cell Culture and MTT Proliferation Assay

Lymph node cells isolated from female C57BL/6 mice were maintained in RPMI 1640 medium supplemented with 100 μgmL^−1^ of streptomycin, 100 UmL^−1^ of penicillin and 10% fetal calf serum under a humidified 5% (v/v) CO_2_ atmosphere at 37 °C. To activate lymph node cells, 5 μgmL^−1^ concanavalin A (Con A) was used. For the MTT proliferation assay, lymph node cells were cultured in 96-well plates either at a density of 3 × 10^5^ cells/well in RPMI 1640 medium (0.2 mL), if stimulated with 5 μgmL^−1^ of Con-A, or at 5 × 10^5^ cells/well in the same medium but without Con-A. After a 48 h treatment with and without the test samples ((−)-, (+)-, and (±)-galewone) at various concentrations, MTT (Sigma; 4 mg mL^−1^ in PBS; 20 mL) was added to each well for a subsequent 4 h incubation. Subsequently, the supernatant was discarded, DMSO (200 mL) was added, and the OD540 was determined by using an ELISA reader (Sunrise, Tecan, Austria).

Immortalized human hepatic stellate cell line LX-2 and activated rat hepatic stellate cell line CFSC-8B were maintained on glass chamber slides in Dulbecco’s modified Eagle’s medium supplemented with 100 μgmL^−1^ of streptomycin, 100 UmL^−1^ of penicillin and 10% fetal calf serum under a humidified 5% (v/v) CO_2_ atmosphere at 37 °C. Cell viability was measured by MTT proliferation assay. LX-2 cells cultured 3 days as quiescent cells^4^.

### Hepatic stellate cells migration assay

The migratory capacity of CFSC-8B was investigated as described before^7^. Specifically, confluent CFSC-8B at the top of BIOCOAT MATRIGEL invasion chamber was incubated in serum-free medium for 24 h. The lower chamber was filled with 10% fetal calf serum in the presence or absence of (−)-galewone at incremental concentrations. Incubating cells for 24 h, then, CFSC-8B from the upper surface of membranes were completely removed with gentle wiping. The migrated cells on the lower surface of membranes were fixed and stained with crystal violet. Migration rate of CFSC-8B was determined by counting the number of stained cells on membranes in six randomly selected fields at high power.

### Hepatic stellate cells wound-healing assay

For determination of cell migration during wound healing, hepatic stellate cells wound-healing assay was measured as described before^5^. CFSC-8B were seeded in 6-wellplates and grown to confluence in Dulbecco’s modified Eagle’s medium containing 10% FBS. Confluent CFSC-8B were deprived of serum for 24 h, and then disrupted to generate a linear wound, followed by incubation in Dulbecco’s modified Eagle’s medium containing 10% FBS in the absence or presence of (−)-galewone for 24 h. Cells were subsequently fixed and observed under phase contrast microscopy, five randomly selected points of each well along each wound were measured.

## Supplementary information

Supporting Information
