# Supplementary material for: Galewone, an Anti-Fibrotic Polyketide from Daldinia eschscholzii with an Undescribed Carbon Skeleton
Source: Sci Rep. 2019 Oct 4;9:14316. doi: 10.1038/s41598-019-50868-9 (PMC6778108; doi:10.1038/s41598-019-50868-9)
Supplement: Supplementary file 1 — Supporting Information [file 41598_2019_50868_MOESM1_ESM.doc]

Supporting Information to

**Galewone, an Anti-Fibrotic Polyketide from *Daldinia eschscholzii* with an Undescribed Carbon Skeleton**

Ai Hua Zhang, Nan Jiang, Xing Qi Wang and Ren Xiang Tan

**Table of Contents**

Supplementary tables . . . . . . . . . . . . . . . . . . . . . . . . . . . . . . . . . . . . . . . . . . . . . . . . 2−5

Supplementary figures . . . . . . . . . . . . . . . . . . . . . . . . . . . . . . . . . . . . . . . . . . . . . . . 6−11

**Table S1**. The IC_50_ values of (−)-, (+)-, and (±)-galewone on CFSC-8B cells.

| Compounds | IC_50_ (μM) |
| --- | --- |
| (–)-galewone | 3.73±0.21 |
| (+)-galewone | 10.10±0.41 |
| (±)-galewone | 10.90±0.62 |
| (–)-galewone ^a^ | 26.60±3.87 |

^a^ IC_50_ value of (–)-galewone on quiescent LX-2 cells.

**Table S2**. ^1^H- and ^13^C-NMR, and HMBC data for galewone in DMSO-*d*_6_.

| position | δ_H_ (multiplicity, *J* in Hz) | δ_C_ | HMBC |
| --- | --- | --- | --- |
| 1 |  | 197.6 |  |
| 2 |  | 124.1 |  |
| 3 |  | 158.0 |  |
| 4 | 6.97 (d, 7.9) | 119.1 | C2, C6 |
| 5 | 7.68 (t, 7.9) | 138.6 | C3, C7 |
| 6 | 7.75 (d, 7.9) | 118.3 | C2, C8 |
| 7 |  | 143.7 |  |
| 8 |  | 139.8 |  |
| 9 |  | 126.6 |  |
| 10 | 8.45 s | 131.6 | C8, C12, C18 |
| 11 |  | 138.7 |  |
| 12 |  | 189.6 |  |
| 13 |  | 114.9 |  |
| 14 |  | 162.0 |  |
| 15 | 7.01 (d, 8.6) | 118.9 | C13, C17 |
| 16 | 7.24 (d, 8.6) | 133.7 | C14, C18, C19 |
| 17 |  | 128.8 |  |
| 18 |  | 132.3 |  |
| 19 |  | 47.5 |  |
| 20 |  | 140.9 |  |
| 21 | 6.54 (d, 7.9) | 117.9 | C19, C23, C25 |
| 22 | 6.99 (t, 7.9) | 135.2 | C20, C24 |
| 23 | 6.77 (d, 7.9) | 117.8 | C21, C25 |
| 24 |  | 162.6 |  |
| 25 |  | 115.1 |  |
| 26 |  | 189.8 |  |
| 27 | 6.94 (d, 10.3) | 132.5 | C19, C25 |
| 28 | 7.48 (d, 10.3) | 158.0 | C20, C26 |
| 29 |  | 65.4 |  |
| 30 |  | 166.7 |  |
| 31 | 4.85 (td, 15.4, 4.0, 1.1) | 60.8 | C10, C12 |
| 3-OH | 8.79 s |  | C2, C3, C4 |
| 14-OH | 12.78 s |  | C13, C14, C15 |
| 24-OH | 12.70 s |  | C23, C24, C25 |
| 30-OCH_3_ | 3.59 s | 53.9 | C30 |

**Table S3. The coordinate information of (+)-galewone.**

|  | X | Y | Z |  |  | X | Y | Z |
| --- | --- | --- | --- | --- | --- | --- | --- | --- |
| C | 0.221 | 3.402 | −1.132 |  | C | −2.800 | −1.738 | −0.797 |
| H | 1.231 | 3.469 | −1.512 |  | C | −1.444 | −1.320 | −0.768 |
| C | −0.693 | 4.414 | −1.455 |  | C | −0.819 | −0.990 | −1.968 |
| H | −0.350 | 5.246 | −2.063 |  | H | 0.225 | −0.711 | −1.984 |
| C | −2.026 | 4.399 | −1.041 |  | C | −1.527 | −1.036 | −3.177 |
| H | −2.710 | 5.196 | −1.312 |  | H | −1.015 | −0.775 | −4.099 |
| C | −2.492 | 3.325 | −0.270 |  | C | −2.860 | −1.407 | −3.219 |
| C | −1.577 | 2.314 | 0.058 |  | H | −3.420 | −1.443 | −4.147 |
| C | −0.222 | 2.331 | −0.346 |  | C | −3.510 | −1.768 | −2.030 |
| C | 0.410 | 1.097 | 0.119 |  | C | −0.070 | 0.515 | 2.445 |
| C | −0.588 | 0.324 | 0.992 |  | C | 0.178 | 2.100 | 4.174 |
| C | −1.895 | 1.137 | 0.837 |  | H | −0.104 | 3.139 | 4.337 |
| C | −0.687 | −1.200 | 0.577 |  | H | 1.262 | 1.982 | 4.221 |
| C | 0.750 | −1.738 | 0.442 |  | H | −0.286 | 1.451 | 4.920 |
| C | 1.836 | −0.896 | 0.145 |  | C | 5.177 | 1.606 | −1.218 |
| C | 1.643 | 0.542 | −0.115 |  | O | −3.771 | 3.268 | 0.132 |
| C | 2.758 | 1.308 | −0.621 |  | H | −3.884 | 2.437 | 0.643 |
| H | 2.631 | 2.363 | −0.812 |  | O | −2.995 | 0.864 | 1.314 |
| C | 4.000 | 0.788 | −0.764 |  | O | −4.669 | −2.585 | 0.406 |
| C | 4.258 | −0.619 | −0.449 |  | O | −4.792 | −2.144 | −2.097 |
| C | 3.134 | −1.451 | −0.024 |  | O | 4.548 | −3.406 | 0.016 |
| C | 3.342 | −2.845 | 0.149 |  | H | 5.176 | −2.667 | −0.213 |
| C | 2.246 | −3.668 | 0.445 |  | O | 5.418 | −1.078 | −0.574 |
| H | 2.413 | −4.734 | 0.556 |  | O | 0.506 | −0.309 | 3.115 |
| C | 0.983 | −3.114 | 0.573 |  | O | −0.305 | 1.774 | 2.856 |
| H | 0.142 | −3.768 | 0.785 |  | O | 4.750 | 2.933 | −1.489 |
| C | −1.404 | −1.942 | 1.691 |  | H | 5.522 | 3.441 | −1.767 |
| H | −0.835 | −2.064 | 2.605 |  | H | 5.617 | 1.126 | −2.106 |
| C | −2.662 | −2.390 | 1.616 |  | H | 5.953 | 1.575 | −0.438 |
| H | −3.146 | −2.878 | 2.456 |  | H | −5.066 | −2.373 | −1.165 |
| C | −3.471 | −2.237 | 0.406 |  |  |  |  |  |

Table S4. TDDFT results for the lowest-energy conformation of (+)-galewone (200 nm < λ < 400 nm). The TDDFT calculations are done at the level of B3LYP/6-31G(d,p) in the PCM model (CH_3_OH solvent: dielectric constant ε = 32.64).

| Transition | Excitation energy (nm) | Rotatory strength *R^a^*  (10^-40^ cgs) | Oscillator strength *f^b^* | Dominant contributions*^c^* | Weight |
| --- | --- | --- | --- | --- | --- |
| 1 | 547.84 | 61.2865 | 0.2098 | 142 →143 | 0.39 |
| 2 | 483.54 | 55.8552 | 0.0543 | 141 →143 | 0.47 |
| 6 | 377.53 | −19.7791 | 0.0955 | 142 →145 | 0.21 |
| 7 | 374.10 | −51.2092 | 0.0535 | 142 →145 | 0.24 |
| 9 | 360.91 | 25.3784 | 0.1503 | 134 →143 | 0.12 |
| 10 | 358.17 | −35.3405 | 0.0751 | 141 →144 | 0.34 |
| 11 | 351.41 | 102.8741 | 0.0590 | 137 →143 | 0.15 |
| 12 | 345.58 | 16.1474 | 0.0051 | 136 →143 | 0.28 |
| 13 | 333.86 | −10.5690 | 0.0085 | 141 →145 | 0.48 |
| 16 | 313.70 | −18.5072 | 0.0071 | 140 →144 | 0.45 |
| 19 | 298.29 | 44.8517 | 0.0173 | 140 →145 | 0.27 |
| 20 | 296.50 | −20.8212 | 0.0135 | 139 →144 | 0.43 |
| 21 | 293.65 | 20.1387 | 0.0245 | 142 →146 | 0.37 |
| 22 | 287.11 | −21.3540 | 0.0117 | 131 →143 | 0.16 |
| 23 | 280.26 | 24.3222 | 0.0310 | 138 →144 | 0.29 |
| 24 | 278.59 | 19.7500 | 0.0161 | 139 →145 | 0.17 |
| 26 | 273.82 | −32.7132 | 0.0374 | 142 →147 | 0.28 |
| 30 | 265.87 | 20.6609 | 0.0221 | 138 →145 | 0.28 |
| 34 | 258.65 | 17.9953 | 0.0052 | 136 →145 | 0.36 |
| 46 | 236.88 | −43.1828 | 0.0981 | 133 →145 | 0.28 |
| 51 | 230.23 | 58.2759 | 0.0265 | 131 →145 | 0.18 |
| 55 | 227.26 | −32.1337 | 0.0739 | 130 →144 | 0.13 |
| 57 | 224.31 | 25.8687 | 0.0913 | 142 →151 | 0.19 |
| 59 | 221.16 | 29.1236 | 0.0996 | 138 →146 | 0.24 |
| 62 | 218.82 | 43.2776 | 0.1271 | 135 →146 | 0.11 |
| 63 | 217.47 | 35.9667 | 0.0156 | 134 →146 | 0.13 |
| 66 | 216.19 | 30.0536 | 0.0970 | 139 →148 | 0.12 |
| 69 | 212.99 | −17.7748 | 0.0123 | 130 →145 | 0.10 |
| 70 | 212.76 | −38.7340 | 0.0292 | 141 →150 | 0.22 |
| 74 | 208.48 | 21.7385 | 0.0626 | 133 →146 | 0.15 |
| 80 | 204.92 | 22.3016 | 0.0625 | 133 →146 | 0.09 |
| 81 | 204.51 | −16.3860 | 0.0142 | 129 →145 | 0.37 |
| 82 | 203.91 | 29.8075 | 0.0424 | 138 →148 | 0.18 |
| 83 | 203.47 | −13.2194 | 0.0042 | 132 →146 | 0.16 |
| 84 | 202.67 | 18.1615 | 0.0388 | 137 →148 | 0.15 |
| 85 | 202.20 | 43.4875 | 0.0363 | 139 →149 | 0.16 |
| 87 | 201.46 | 36.5117 | 0.0574 | 132 →146 | 0.08 |
| 88 | 200.73 | −84.5985 | 0.1192 | 139 →149 | 0.12 |
| 89 | 200.23 | 18.6449 | 0.0140 | 139 →149 | 0.09 |

^a^ All the strengths were in the velocity representation.

^b^ Excited states with f < 0.1 and R < ± 10.0 were not presented.

^c^ Configurations with weights below 0.10 were not displayed.


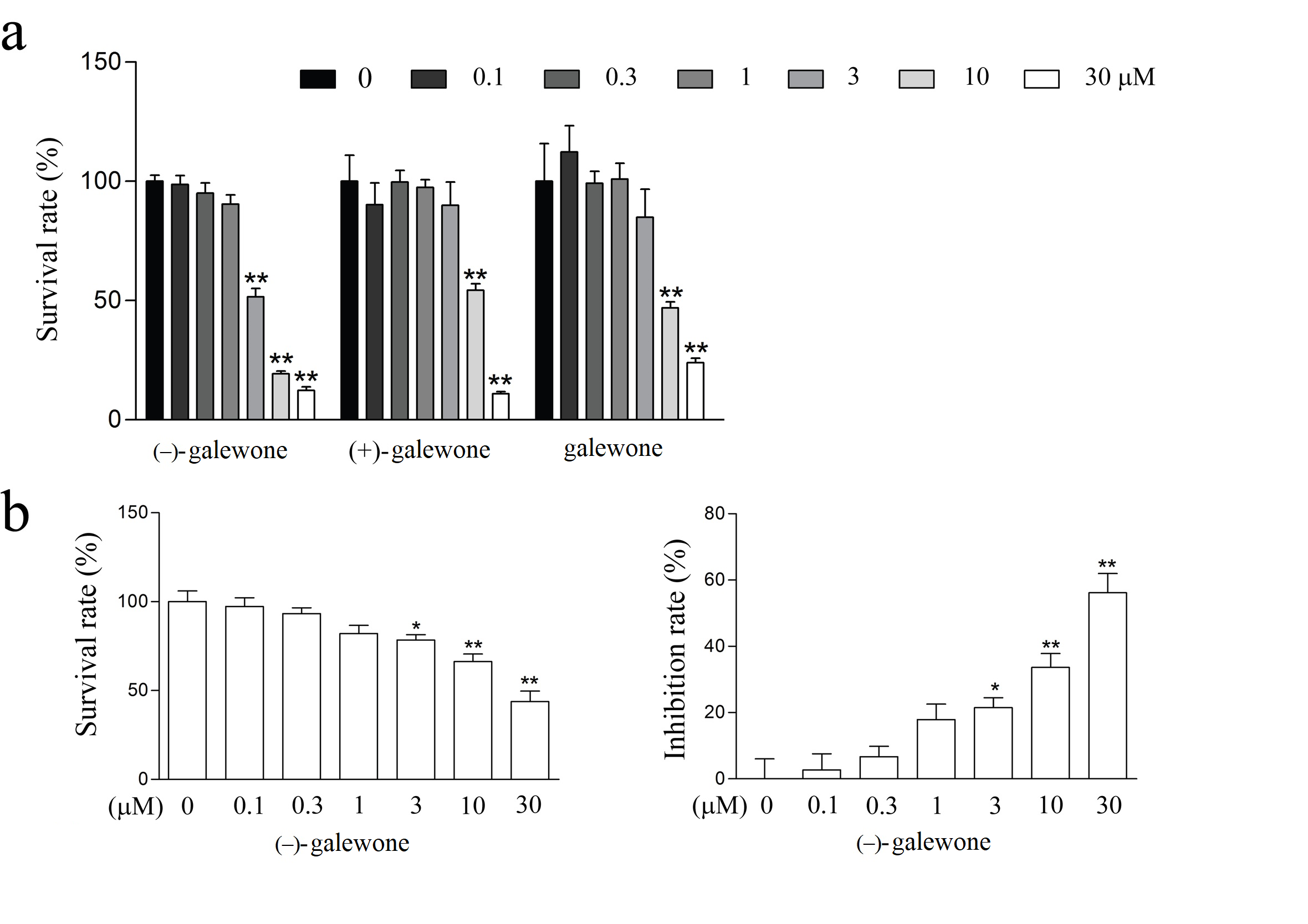


**Figure S1.** The inhibition of (–)-galewone, (+)-galewone and galewone on the proliferation of CFSC-8B cells (**a**), and the effects of (–)-galewone on the survival of quiescent LX-2 cells (**b**). All data represent the mean±SEM of three independent experiments in triplicate. **P* < 0.05, ***P* < 0.01 *vs.* vehicle group.


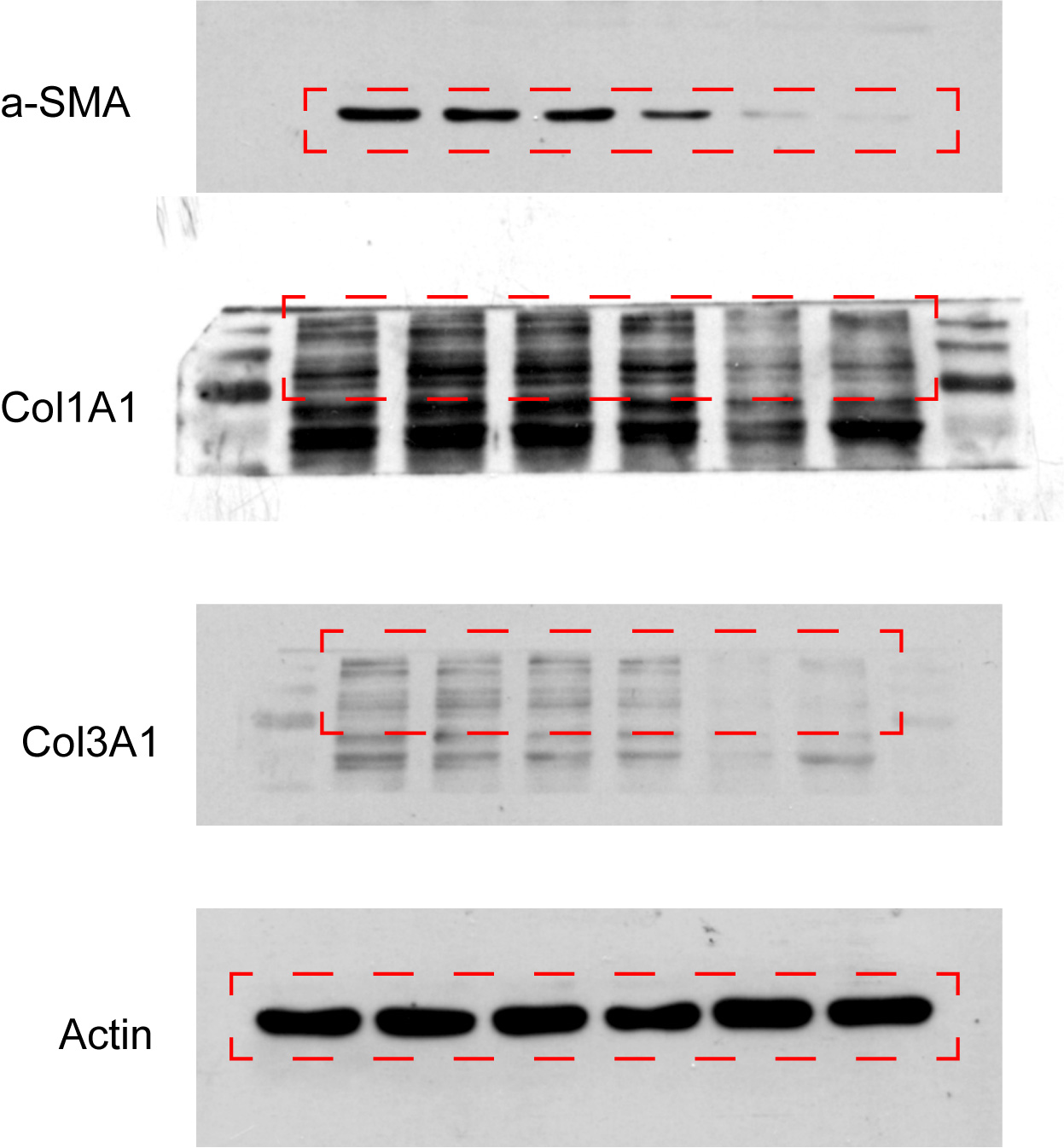


**Figure S2**. Full length western blots in figure 4d and the target protein bands were marked by red box.

(+)-galewone

(–)- galewone

<Column Performance Report>

Column: CHIRALPAK® IA^TM^, 0.50 cm ID. × 25 cm L, 5 µm

Mobile Phase: DCM/MeOH = 10/90 (v/v)

Peak No. Time Area Area %

1 9.141 2577.11 49.0892

2 10.994 2599.68 49.5190

**Figure S3.** Chiral HPLC separation of galewone


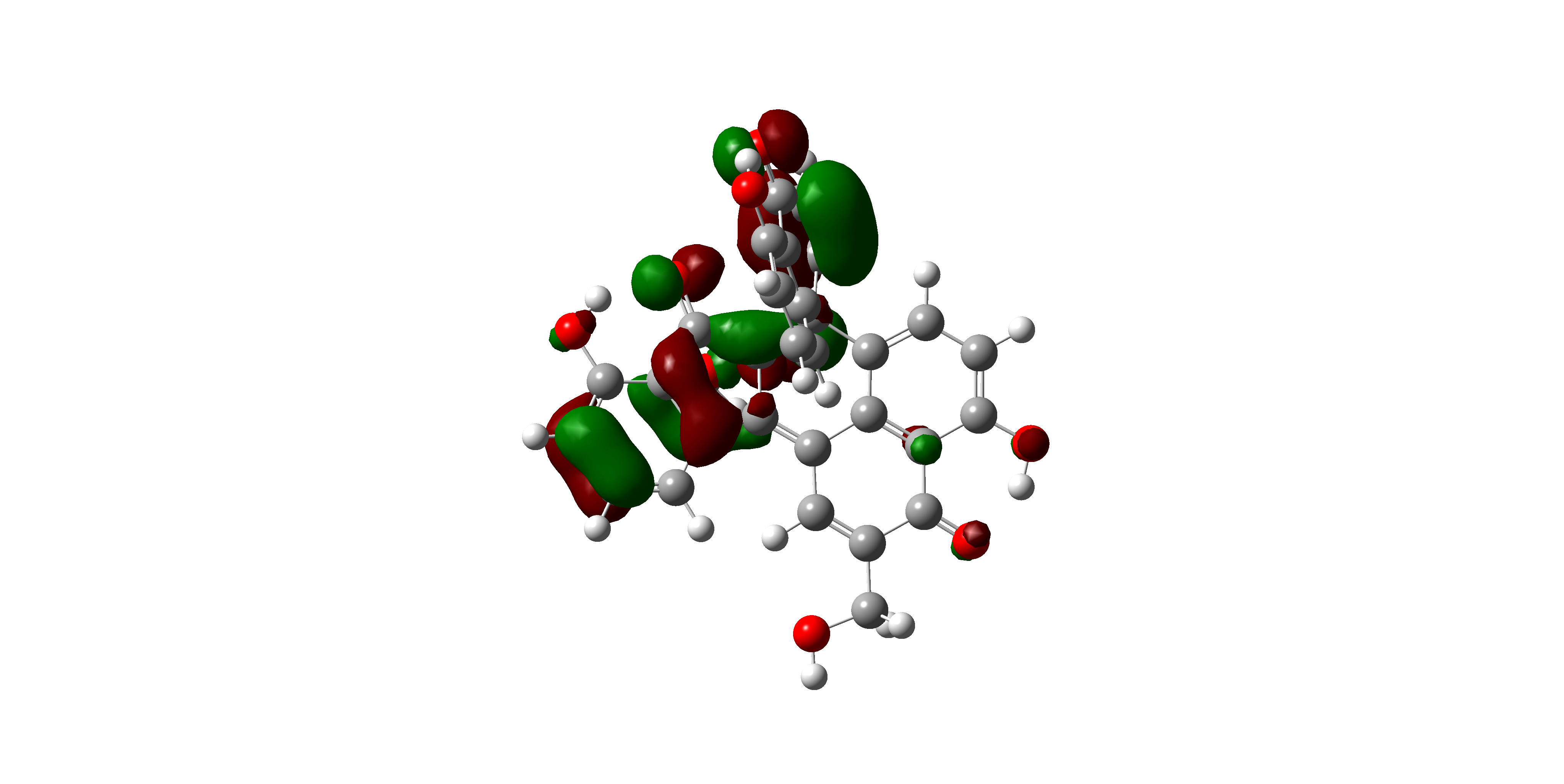

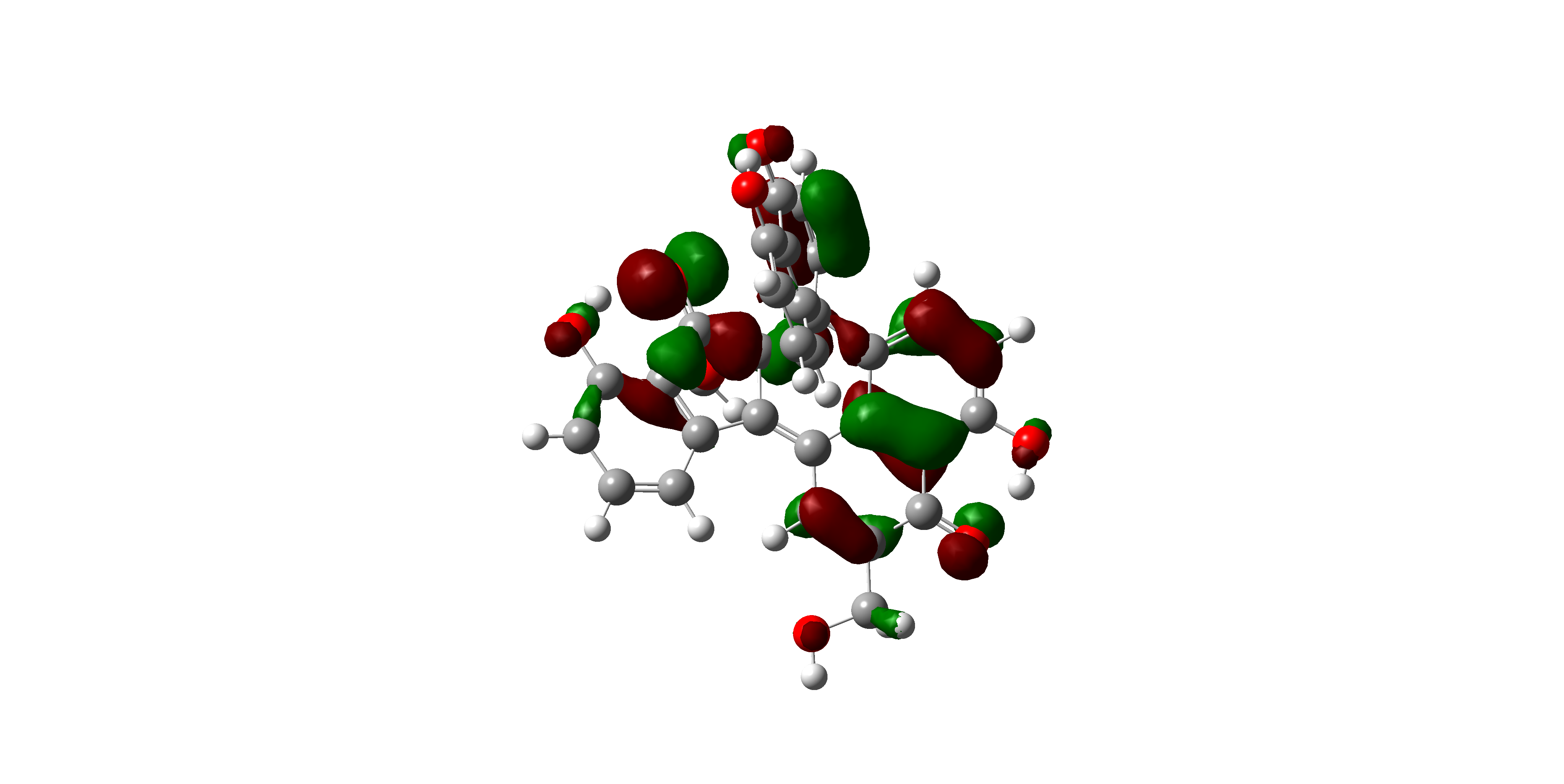

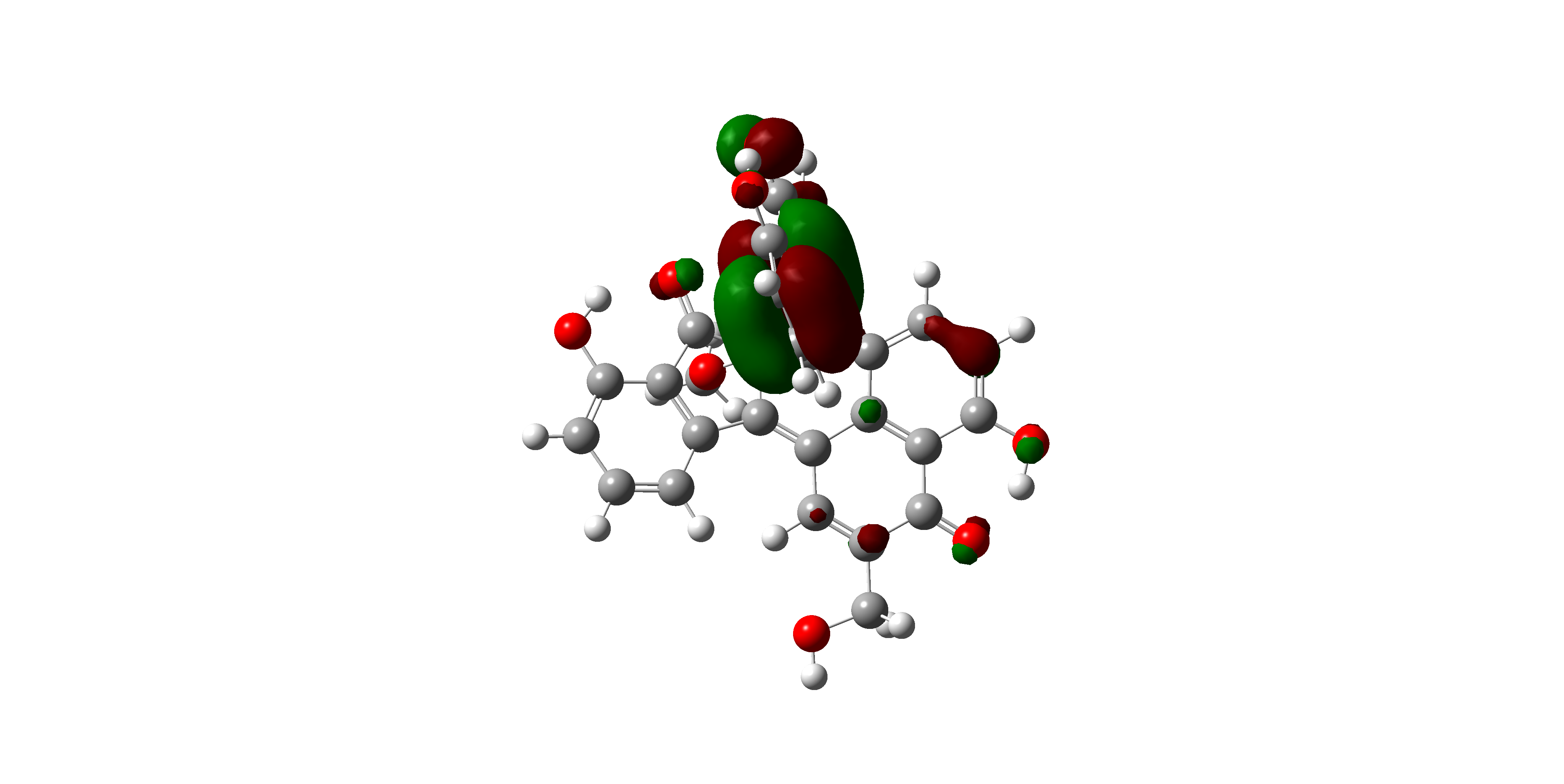

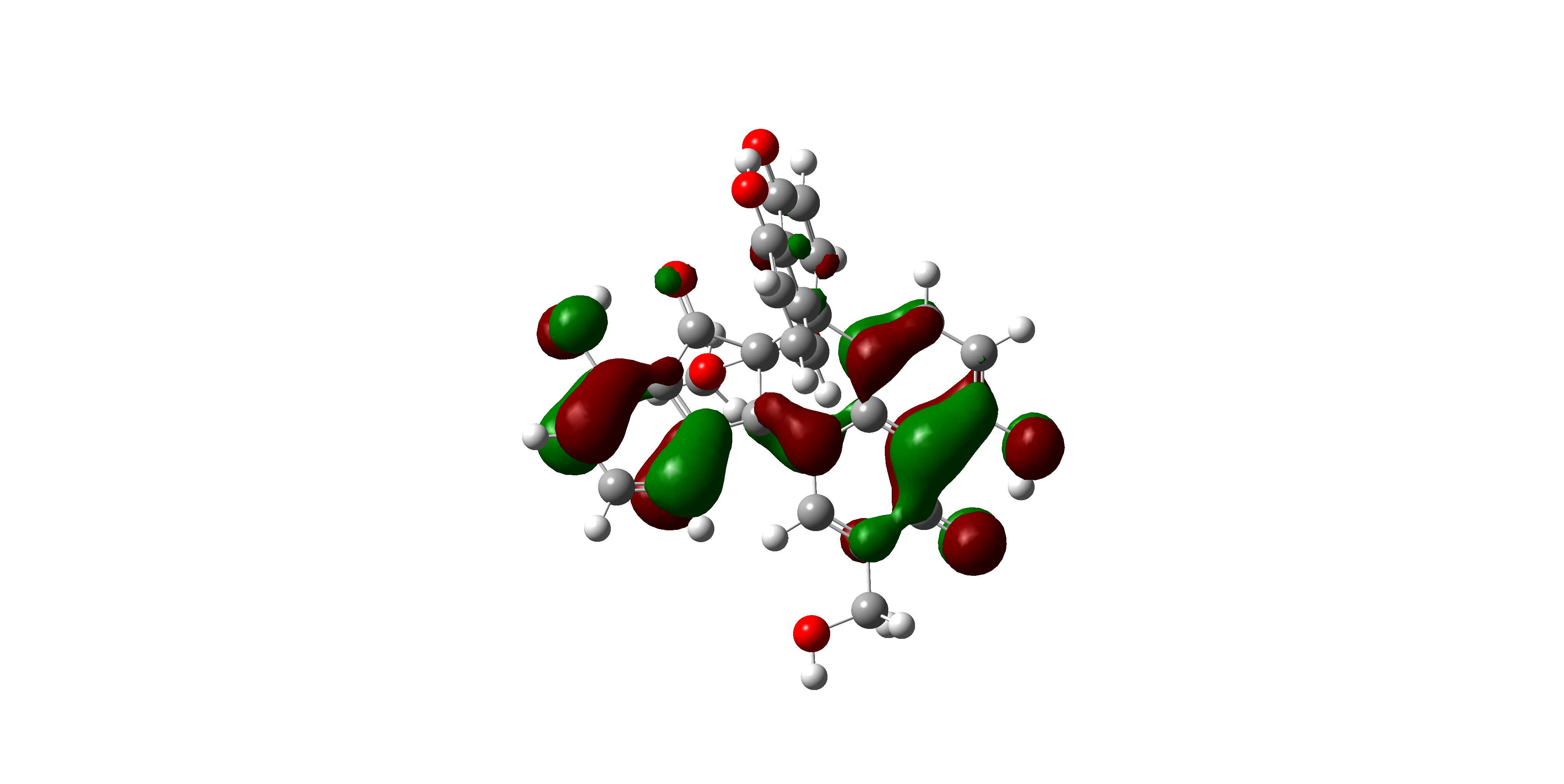


MO 133 (‒7.70 eV) MO 135 (‒7.26 eV) MO 137 (‒7.10 eV) MO 140 (‒6.58 eV)


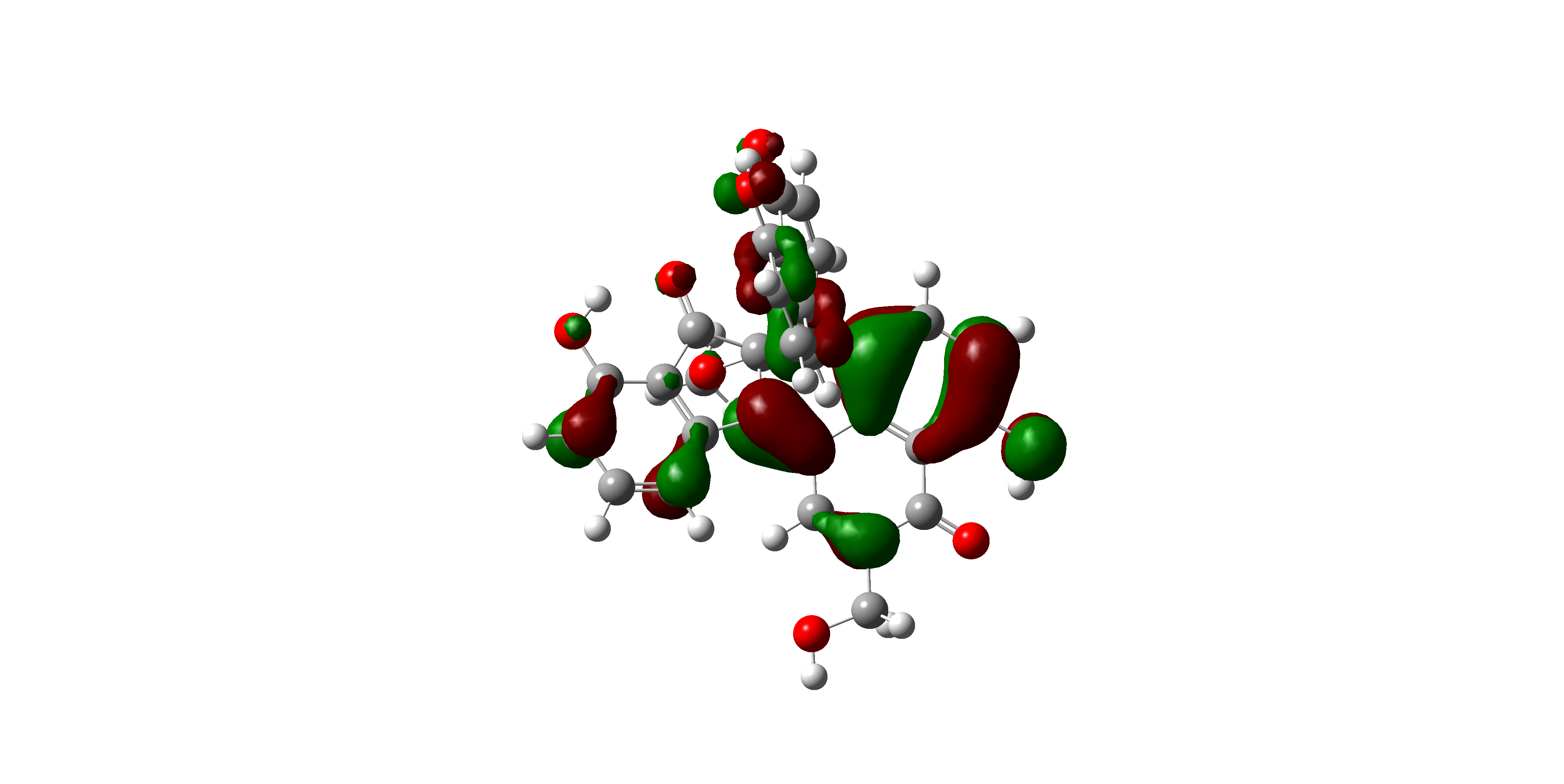

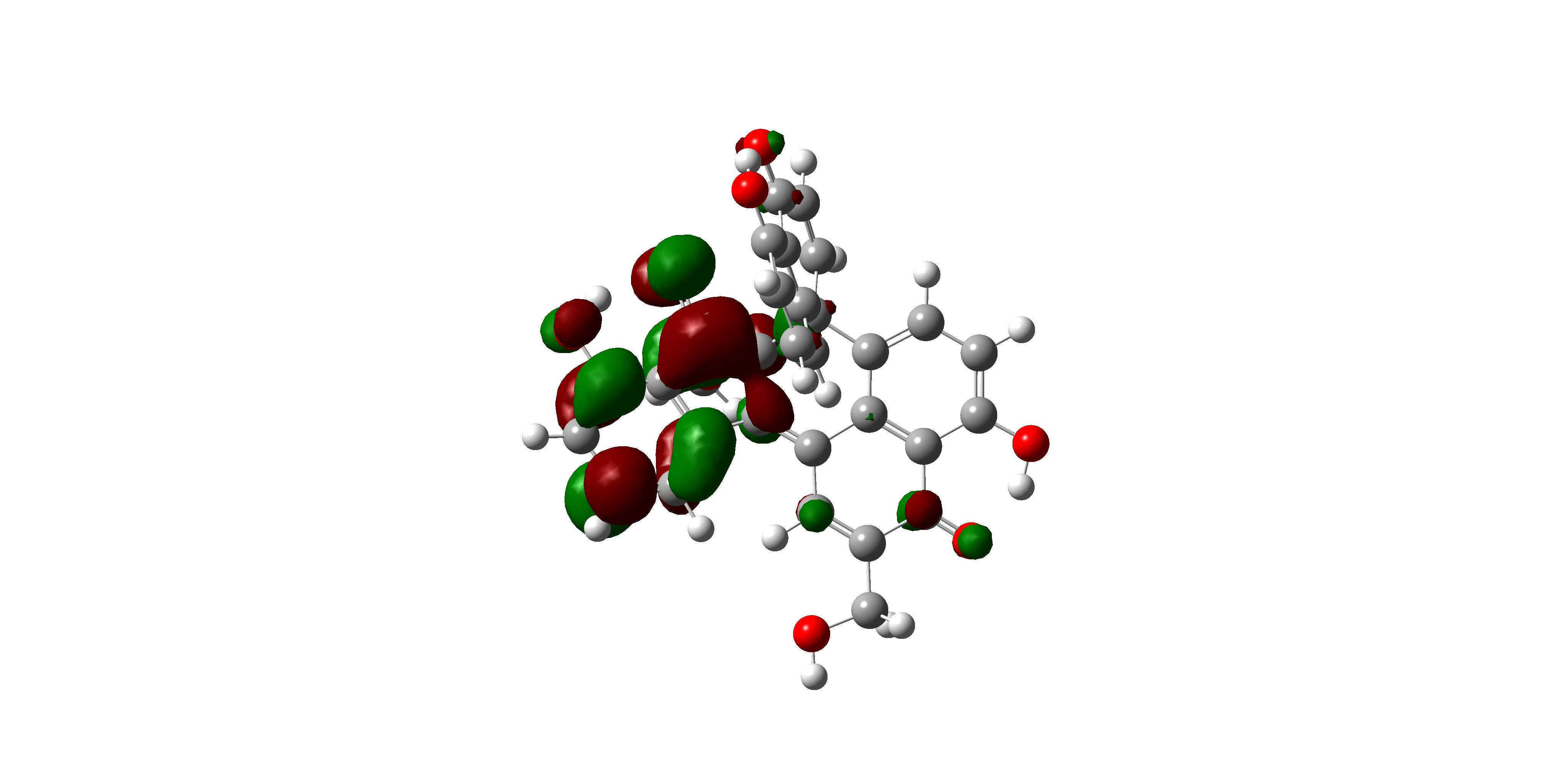

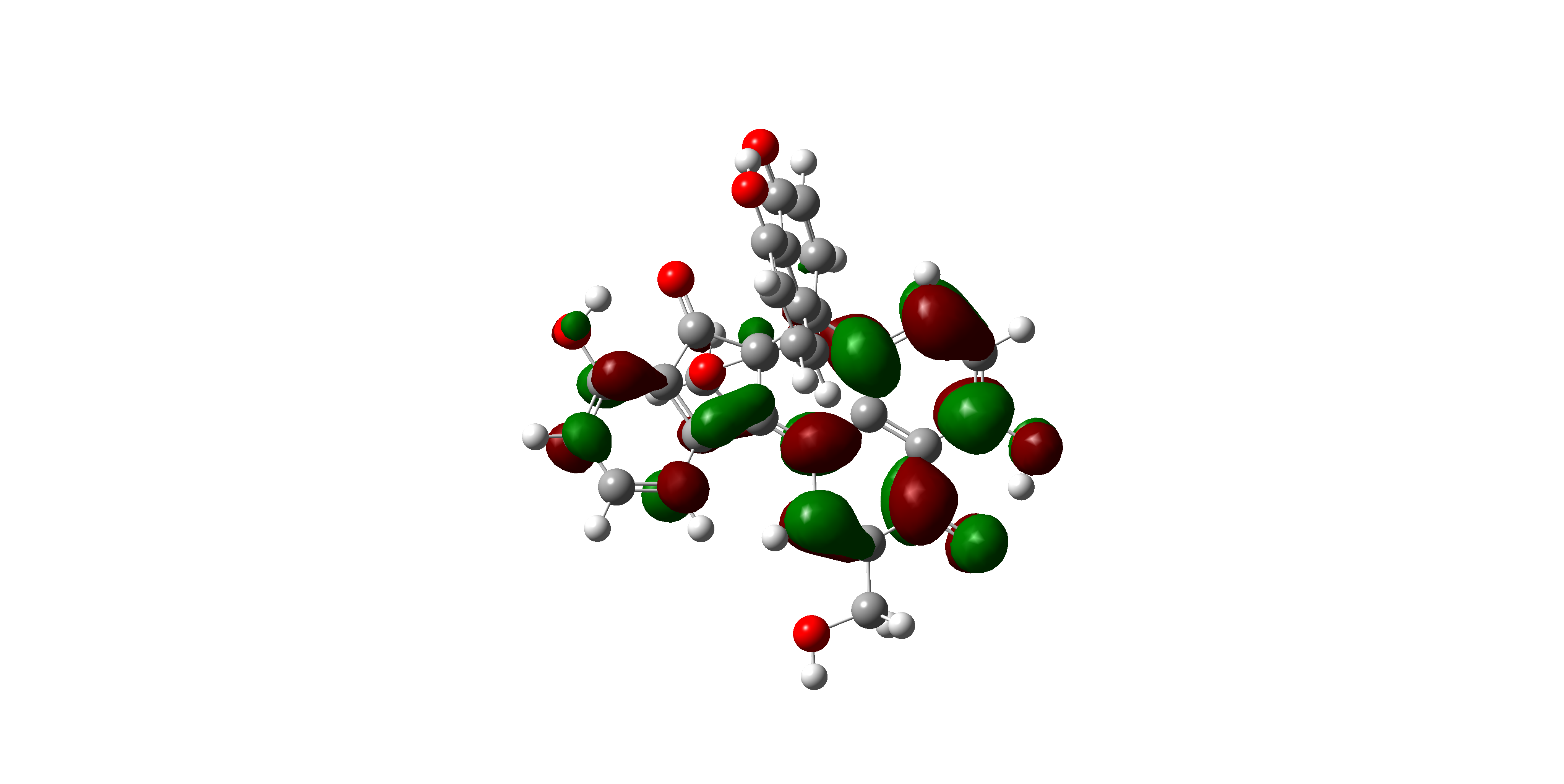


MO 142 (‒5.77 eV) MO 145 (‒1.93 eV) MO 146 (‒1.03 eV)

**Figure S4.** The important molecular orbitals (MOs) of the optimized conformer of (‒)-galewone.


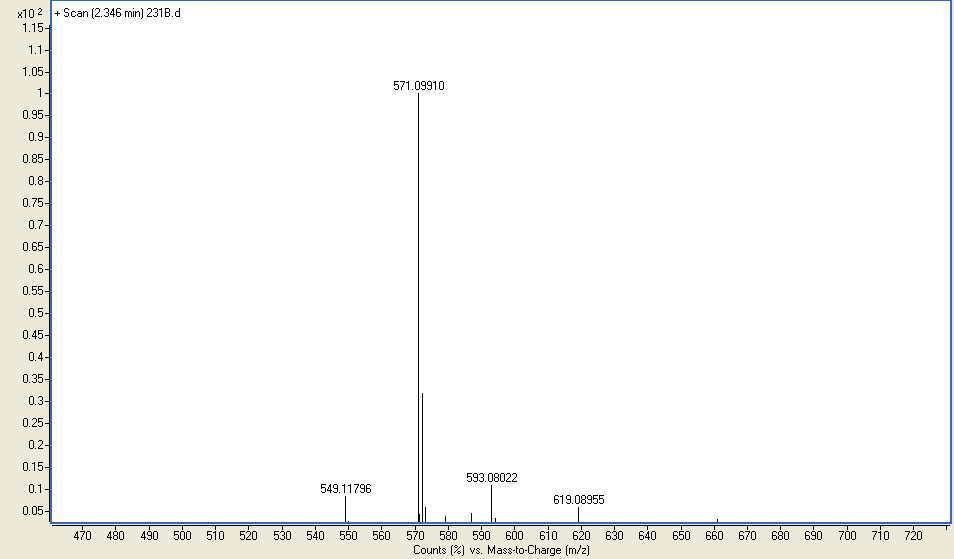


**Figure S5**. HR-ESI-MS of galewone in CD_3_OD


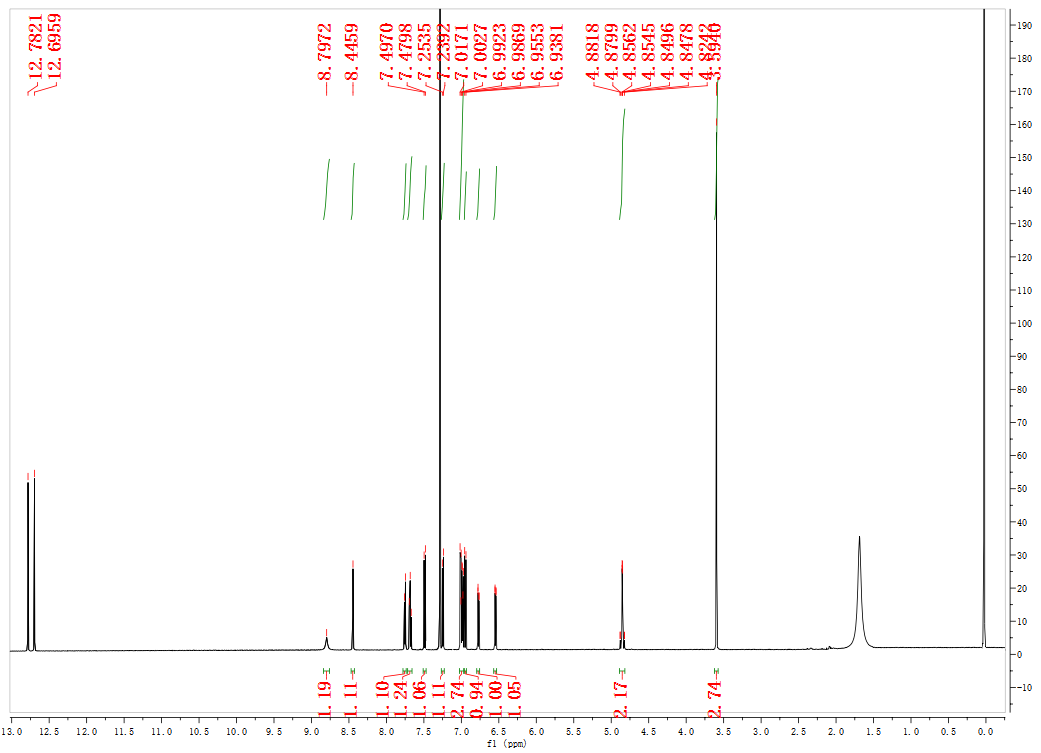


**Figure S6**. ^1^H NMR spectrum of galewone (600 MHz, CDCl_3_).

**
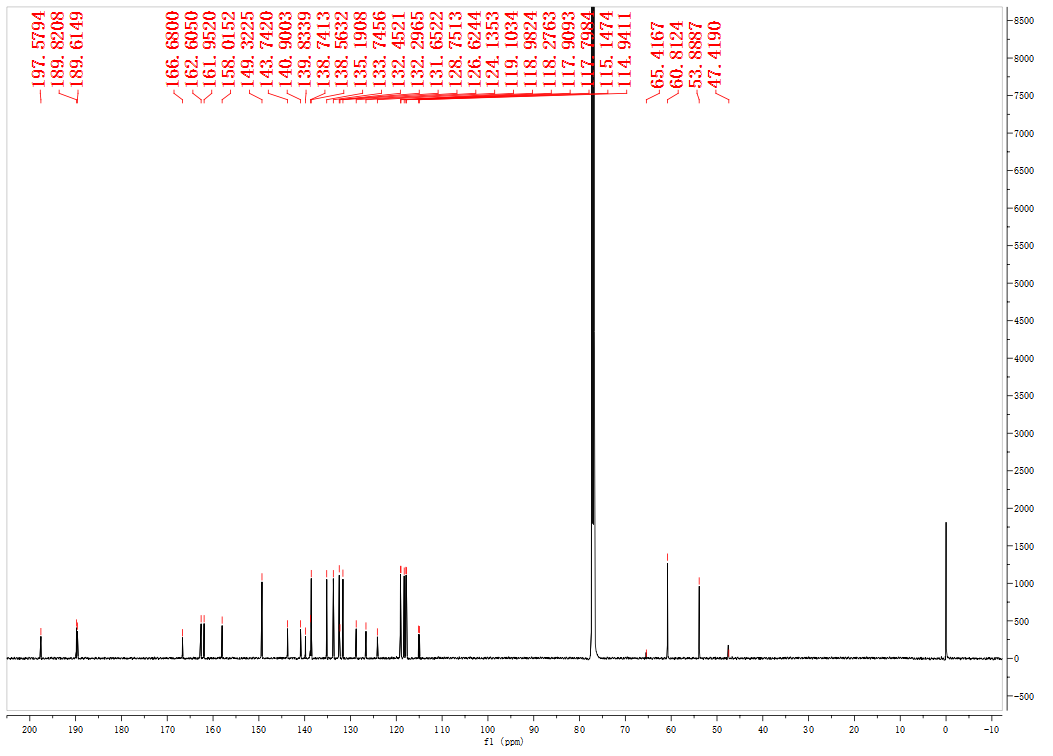
**

**Figure S7**. ^13^C NMR spectrum of galewone (150 MHz, CDCl_3_).

**
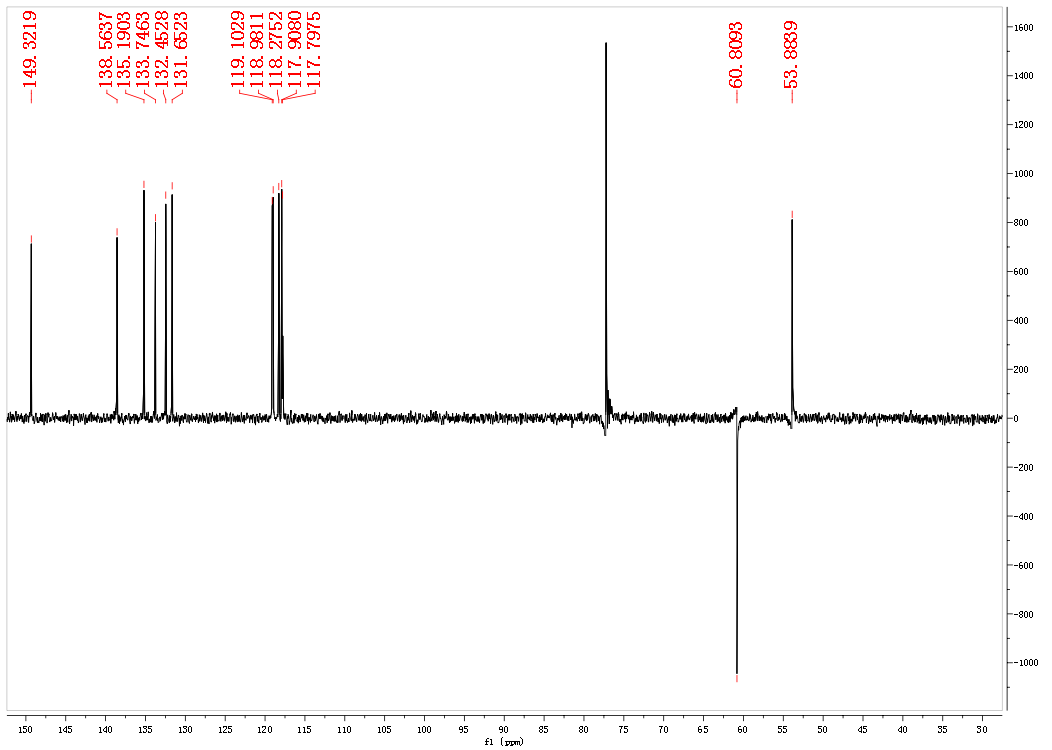
**

**Figure S8**. DEPT spectrum of galewone (CDCl_3_).

**
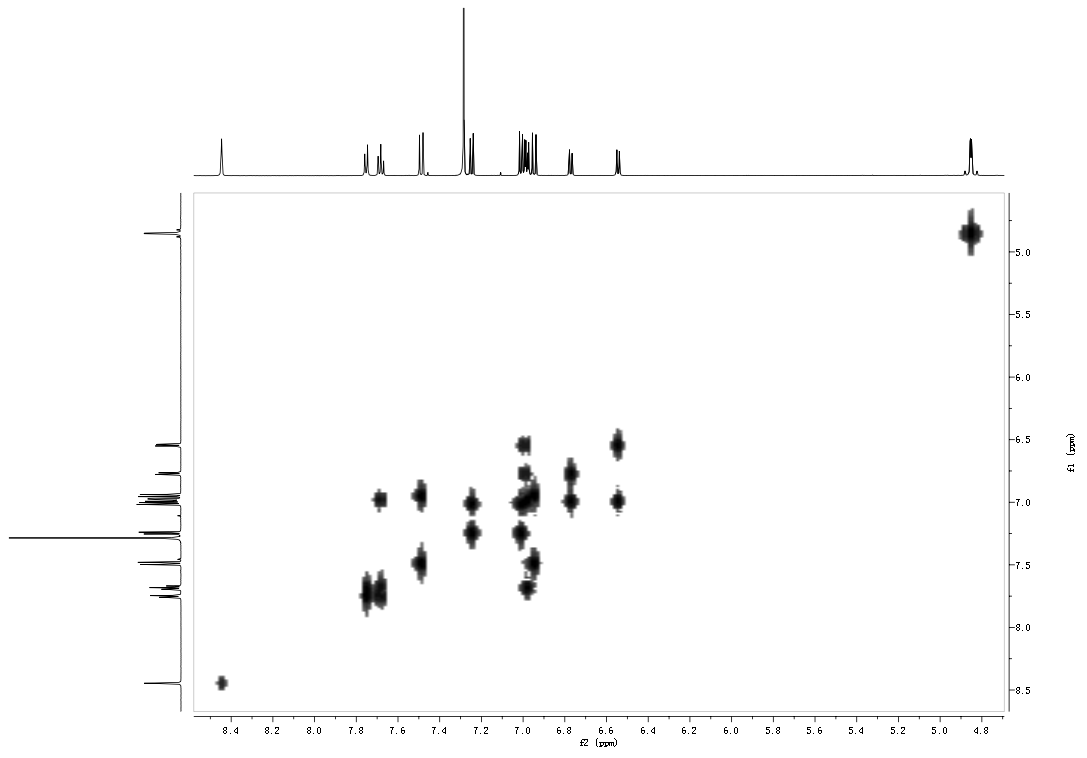
**

**Figure S9**. ^1^H-^1^H COSY spectrum of galewone (CDCl_3_).


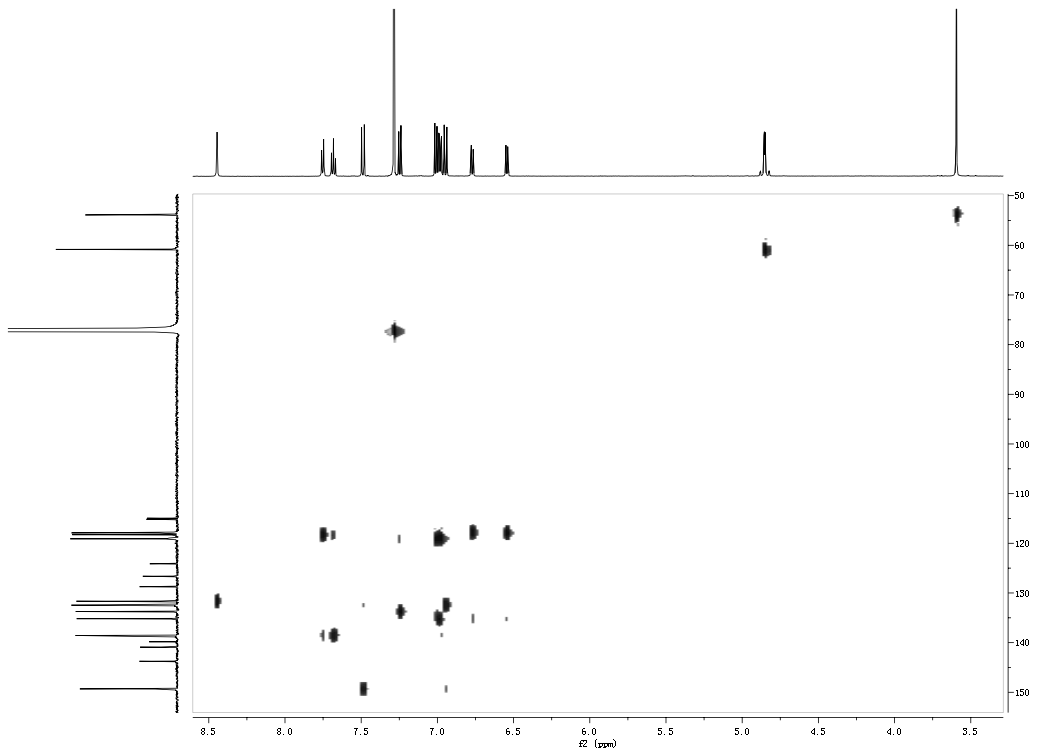


**Figure S10**. HSQC spectrum of galewone (CDCl_3_).


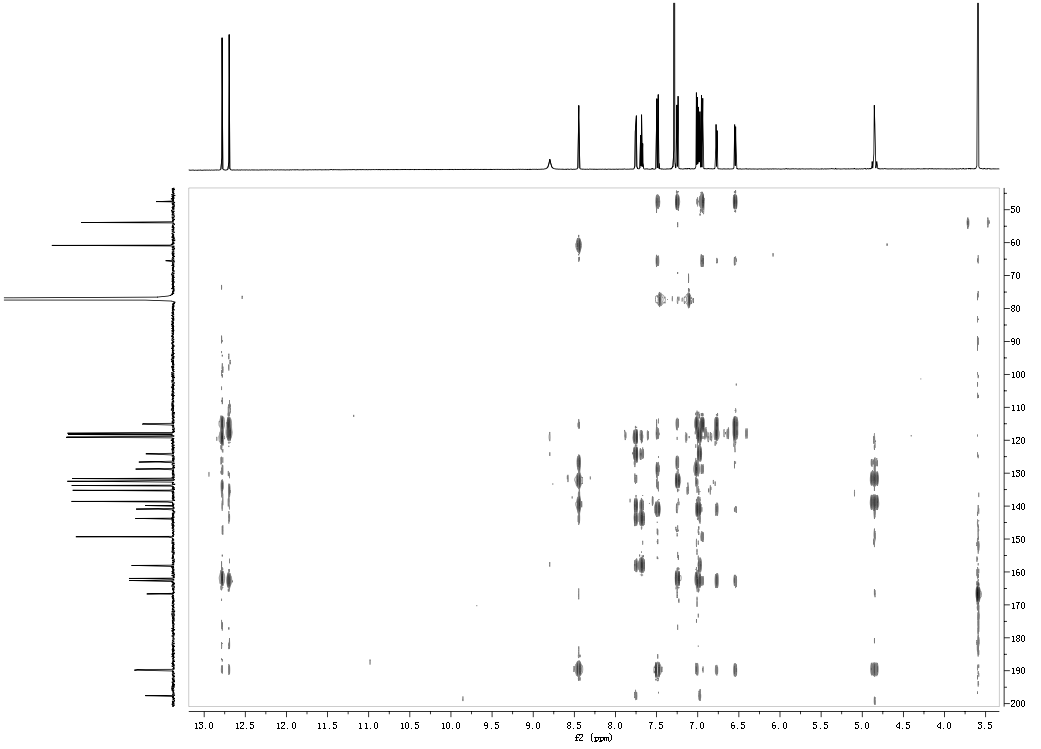


**Figure S11**. HMBC spectrum of galewone (CDCl_3_).
